# Spinal fusion for single-level SPECT/CT positive lumbar degenerative disc disease: the SPINUS I study

**DOI:** 10.1007/s00701-023-05666-8

**Published:** 2023-06-22

**Authors:** Radek Kaiser, Michal Varga, Otto Lang, Petr Waldauf, Petr Vaněk, Karel Saur, Vladimír Beneš, David Netuka

**Affiliations:** 1 Department of Neurosurgery and Neurooncology, First Faculty of Medicine, Charles University and Military University Hospital Prague, U Vojenské Nemocnice 1200, 16902 Prague, Czech Republic; 2 Department of Spinal Surgery, First Faculty of Medicine, Charles University and University Hospital Motol, Prague, Czech Republic; 3 Department of Nuclear Medicine, Third Faculty of Medicine, Charles University and University Hospital Kralovske Vinohrady, Prague, Czech Republic; 4 Department of Anaesthesia and Intensive Care Medicine, Third Faculty of Medicine, Charles University and University Hospital Kralovske Vinohrady, Prague, Czech Republic

**Keywords:** Spinal fusion, Low back pain, SPECT, Degenerative disc disease, Axial pain

## Abstract

**Introduction and purpose:**

With current imaging modalities and diagnostic tests, identifying pain generators in patients with non-specific chronic low back pain (CLBP) is difficult. There is growing evidence of the effectiveness of SPECT/CT examination in diagnosing the source of pain in the spine. The study aims to investigate the effect of posterior interbody fusion on a single-level SPECT/CT positive lumbar degenerative disc disease (DDD).

**Material and methods:**

This is a prospective study of patients with chronic low back pain (CLBP) operated on for a single-level SPECT/CT positive DDD. Primary outcomes were changes in visual analogue scale (VAS) scores and the Oswestry Disability Index (ODI). Secondary outcomes were complications, return to work, satisfaction and willingness to re-undergo surgery.

**Results:**

During a 3-year period, 38 patients underwent single-level fusion surgery. The mean preoperative VAS score of 8.4 (± 1.1) decreased to 3.2 (± 2.5, *p* < 0.001) and the mean preoperative ODI of 51.5 (± 7.3) improved to 20.7 (± 14.68, *p* < 0.001) at a 2-year follow-up. A minimum clinically important difference (30% reduction in VAS and ODI) was achieved in 84.2% of patients. Some 71% of patients were satisfied with the surgery results and 89.4% would undergo surgery again. There were four complications, and two patients underwent revision surgery. Some 82.9% of patients returned to work.

**Conclusion:**

Fusion for one-level SPECT/CT positive lumbar DDD resulted in substantial clinical improvement and satisfaction with surgical treatment. Therefore, SPECT/CT imaging could be useful in assessing patients with CLBP, especially those with unclear MRI findings.

**Trial registration:**

ClinicalTrials.gov Identifier: NCT04876586.

**Supplementary information:**

The online version contains supplementary material available at 10.1007/s00701-023-05666-8.

## Introduction

Chronic low back pain (CLBP) caused by spinal degeneration is a common clinical condition, with prevalence reaching up to 58% in adult populations [13]. Degenerative disc disease (DDD) or facet osteoarthritis is most frequently considered a pain generator where significant disc herniation, spinal stenosis, instability or deformity is excluded. However, identifying the source of pain in these patients is challenging because of the high incidence of such changes in the general population without a painful correlate [16] and the controversial role of invasive tests in detecting pain generators [28].

Fusion surgery in such cases is in dispute. The National Institute for Health and Care Excellence (NICE) has issued guidelines advising that fusion for non-specific CLBP should only be performed as part of a clinical trial and that lumbar disc replacement should not be performed [23].

Radionuclide bone scintigraphy with single photon emission computed tomography (SPECT) provides functional imaging of increased osteoblastic activity representing areas of mechanical stress and degenerative changes in the skeleton. Multimodality SPECT/computed tomography (CT) allows the high sensitivity of SPECT to be combined with the specificity and higher spatial resolution of CT [17]. Increasing evidence exists on the relationship between pain-causing DDD or facet arthropathy and SPECT positivity [6, 24]. These findings have been recently confirmed by surgical studies [3, 21]. However, the evidence for the effect of surgical treatment of SPECT-positive lumbar degeneration is not conclusive because of the small number of operated cases, combining different surgical techniques and single- and multi-level fusions in different areas of the spine [25].

This study aimed to evaluate the effect of posterior interbody fusion on a single-level SPECT/CT positive lumbar DDD.

## Material and methods

This study is a prospective analysis of patients who underwent bone SPECT/CT and surgery for a single-level SPECT/CT positive DDD (SPECT + DDD) at our institution between January 2018 and December 2020. The study was approved by our institutional research board. All patients signed an informed consent form before entering the study. The study was performed in accordance with the ethical standards of the 1975 Declaration of Helsinki and its amendments of 2013. ClinicalTrials.gov Identifier: NCT04876586.

The SPECT/CT imaging was offered to patients who fulfilled the following criteria:

- Age between 18 and 75 years
- Severe CLBP (> 5 points on the visual analogue scale, VAS) with duration > 1 year
- Failure of non-surgical treatment lasting minimally 6 months combining analgesia and physical therapy
- Radiological evidence of multi-level spinal degeneration (DDD and/or facet arthropathy) on lumbar magnetic resonance imaging (MRI) without evidence of significant disc herniation, spinal stenosis or instability (confirmed on flexion/extension X-ray) corresponding to clinical symptoms

Exclusion criteria:

- Other spinal pathology (tumours, congenital defects, trauma, inflammation, deformity)
- Previous surgical stabilisation surgery of the spine
- Intolerance to SPECT examination
- Psychiatric illness or evidence of emotional instability
- Pregnancy

### Bone scan imaging protocol

**Fig. 1:**
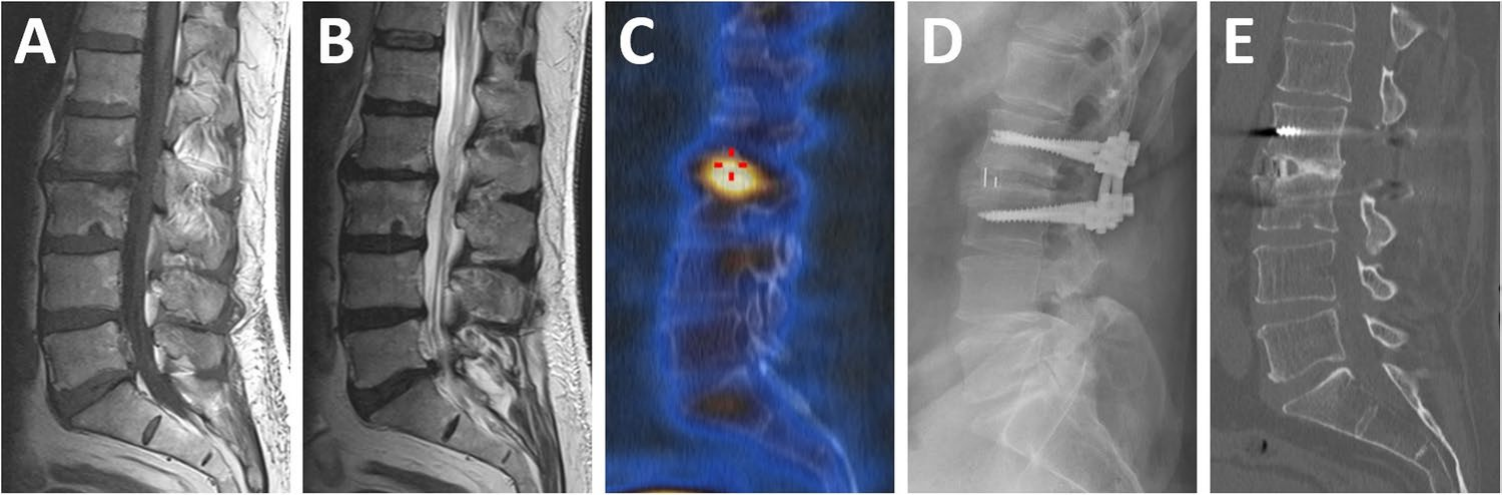
Multi-level degeneration (patient 30) seen on sagittal lumbar spine images. **A** T1W MRI, **B** T2W MRI, **C** SPECT/CT showing higher uptake in L2/3 level, **D** X-ray 6 months after surgery, **E** CT scan 2 years after surgery with fusion of the L2/3 intervertebral space

Three-phase bone scan protocol was used in all patients. 99mTc hydroxy diphosphonate (HDP) was administered intravenously (10 MBq/kg), and immediate angiographic phase and blood pool planar images were obtained over the lumbar spine. After a 2–3 h interval, a whole-body bone and a SPECT/CT scan of the lumbar spine were performed (GE dual-head gamma camera Optima NM/CT 640). SPECT data were acquired with a low-energy high-resolution collimator (128 × 128 matrix with body contouring). Non-diagnostic CT scans were obtained at fixed 120 kVp with 30 mAs. The SPECT data were reconstructed using an iterative reconstruction algorithm. The reconstructed attenuation-corrected SPECT data were co-registered with CT data and viewed in multiplanar projections. Sites of increased tracer uptake were considered positive for DDD or facet arthropathy according to localisation detected by CT (Fig. 1).

### Surgical treatment

Fusion surgery of the SPECT + DDD lumbar segment was performed by an open TLIF (transforaminal lumbar interbody fusion) procedure [18]. The control CT scan of the lumbar spine was performed on the first postoperative day. Immediately thereafter, the patient was allowed to stand, walk and undergo physiotherapy. Patients were discharged to home care within 5–7 days after surgery.

### Collected data and outcome measures

We assessed sociodemographic and clinical characteristics. Pain, disability and complications were measured at baseline and 6 and 24 months after surgery. Evaluation of questionnaires, return to work, satisfaction and willingness to undergo surgery again were evaluated by a PhD candidate (MV) not involved in the treatment process.

#### Primary outcomes

- Back pain intensity was measured on a vertical VAS [14], ranging from 0 to 10, where 10 indicated the worst pain imaginable.
- Pain-related disability was measured by the Oswestry Disability Index (ODI) version 2.1a [8].
- A 30% reduction from baseline in both VAS and ODI was set as the minimum clinically important difference (MCID) [1].

#### Secondary outcomes

- Surgery complications, postoperative complications, implant failure and repeat surgery
- Return to work
- Satisfaction with the effect of surgery and willingness to undergo a new surgery, both estimated on a 5-point scale (1 = definitely, 2 = rather yes, 3 = maybe, 4 = rather no, 5 = definitely not)

### Statistics

All analyses were performed using the statistical software R version 4.2.2. Exploratory data analysis was performed for all parameters. Continuous data are reported as mean ± SD or median and interquartile range as appropriate. A mixed-effect model with a random intercept was used to compare the preoperative and two postoperative scores. Linear regression was conducted to compare the effect of the other parameters (age, sex, body mass index (BMI) level, satisfaction and willingness to undergo re-operation) on the decrease in scores before surgery and at the follow-up visits. A *p*-value < 0.05 was considered statistically significant.

## Results

During a 3-year period, we performed 139 SPECT/CT examinations in patients with CLBP. No active bone metabolism was detected in 50 cases (36%). Isolated sacroiliitis was diagnosed in three patients (3%). Multi-level SPECT + DDD was seen in 15 patients and facet arthropathy with or without DDD in 29. Isolated one-level SPECT + DDD was detected in 42 patients. However, four patients did not undergo surgery because of a preference to continue conservative therapy.

Thirty-eight patients underwent single-level TLIF for SPECT + DDD. Five patients had a history of previous microdiscectomy in the affected segment (L5/S1 in all cases), while 33 never underwent surgery in the lumbosacral region. Data on participant characteristics and types of surgery are summarised in Table 1. There were 25 female (F) and 13 male (M) patients (F:M ratio 1.9:1) ranging in age from 27 to 75 years (mean 48.05 ± 10.5 years) and with BMI between 19.7 and 38.6 (mean 26.7 ± 4.6).

**Table 1:** Patient characteristics. * statistically significant association with a lower decrease in VAS after surgery (*p* < 0.05)

Age	48.05 (± 10.5)	
BMI	26.7 (± 4.6)	
Sex	Males	13 (34.2%)
	Females	25 (65.8%)
Spinal level	L1/2	1 (2.6%)
	L2/3	3 (7.9%)
	L3/4	2 (5.3%)
	L4/5	6 (15.8%)
	L5/S1	26 (68.4%)
VAS pre-OP	8.4 (± 1.1)	
VAS 6 months post-OP	3.3 (± 2.5, p < 0.001)	
VAS 24 months post-OP	3.2 (± 2.5, p < 0.001)	
ODI pre-OP	51.5 (± 7.3)	
ODI 6 months post-OP	20.9 (± 14.96, p < 0.001)	
ODI 24 months post-OP	20.7 (± 14.68, p < 0.001)	
MCID	32 (84.2%)	
Satisfaction with the effect of surgery	1—definitely	19 (50%)
	2—rather yes	8 (21%)
	3—maybe	6 (15.8%)
	4—rather no	4 (10.5%) *
	5—definitely not	1 (2.7%) *
Willingness to undergo surgery again	1—definitely	30 (78.9%)
	2—rather yes	4 (10.5%)
	3—maybe	0 (0%)
	4—rather no	2 (5.3%)
	5—definitely not	2 (5.3%) *
Sick leave pre-OP	19 (50%), mean 8 (± 4) months	
Sick leave post-OP	33 (86.8%), mean 6 (± 5.6) months	
Disability pension pre-OP	2 (5.3%)	
Disability pension post-OP	6 (15.8%)	
Maternity leave pre-OP	1 (2.6%)	
Old-age pension pre-OP	2 (5.3%)	
Early complications	3 (7.9%)	
Revision surgery	2 (5.3%, one revised twice)	

**Fig. 2:**
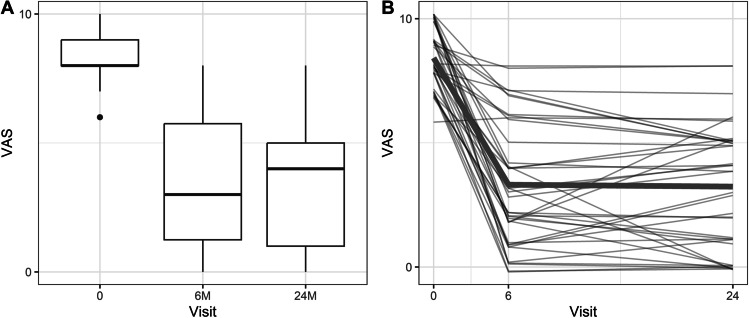
**A** Box plot showing VAS at baseline and 6 and 24 months after surgery, **B** Graph depicting the change of VAS over time in individual patients, wide line – mean change

**Fig. 3:**
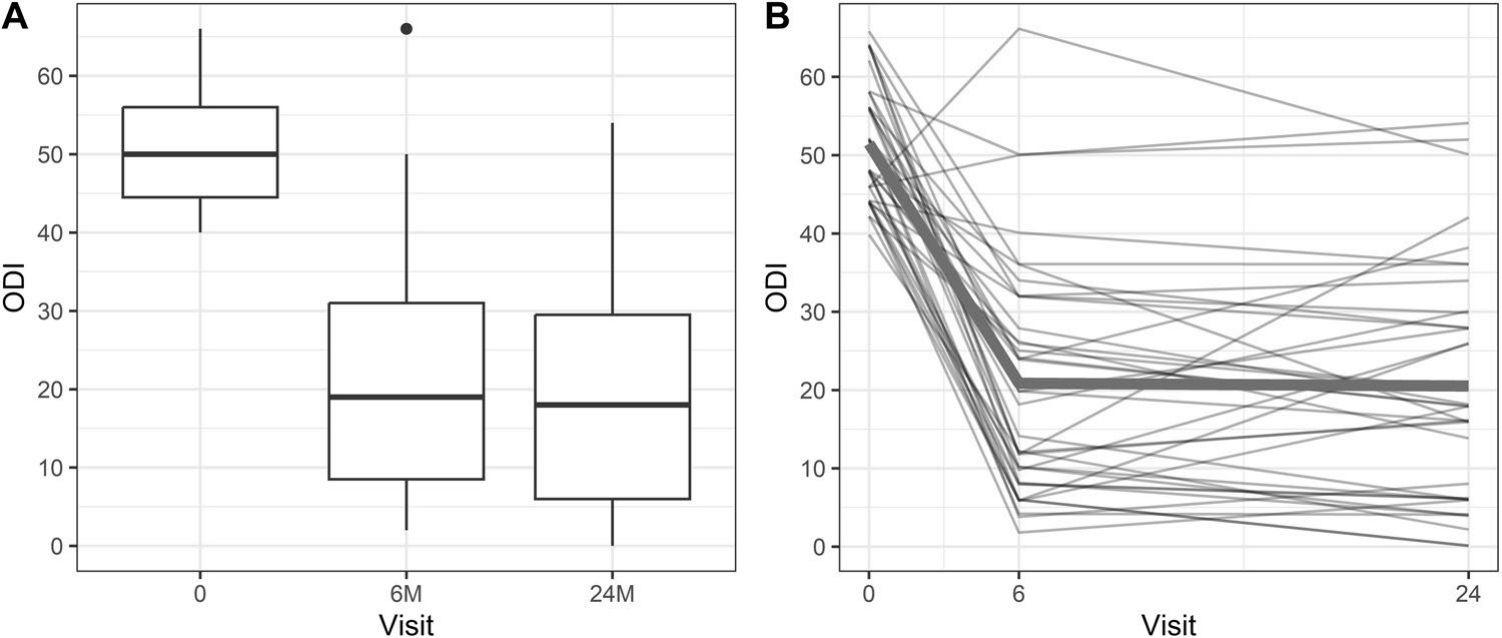
**A** Box plot depicting ODI at baseline and 6 and 24 months after surgery, **B** Graph illustrating the change of ODI over time in individual patients, wide line – mean change

The mean preoperative VAS score of 8.4 (± 1.1) significantly decreased at 6 months to 3.3 (± 2.5, *p* < 0.001) and 3.2 at 24 months (± 2.5, *p* < 0.001, Fig. 2). Mean preoperative ODI was 51.5 (± 7.3), and it also improved significantly at 6- and 24-month follow-ups to 20.9 (± 14.96, *p* < 0.001) and 20.7 (± 14.68, *p* < 0.001, Fig. 3), respectively. MCID was achieved in 32 (84.2%) patients. The difference in VAS (*p* = 0.88) and ODI (*p* = 0.83) between the 6- and 24-month follow-ups did not reach statistical significance. The change in VAS and ODI was not influenced by age, sex, BMI and the operated level (*p* > 0.05). Patient-level data are presented in the Appendix.

Early adverse events occurred in three (7.9%) patients: (1) deep infection with revision surgery, (2) two revision surgeries due to CSF leak followed by wound infection and sepsis and (3) painful subcutaneous swelling around the scar developed a few weeks after surgery with negative MRI, allergy and immunological tests. Another patient developed symptomatic adjacent segment disease 18 months after surgery (treated conservatively).

Three patients (one on maternity leave and two on old-age pension) were excluded from the return-to-work analysis. Of the remaining 35 patients, 29 (82.9%) returned to work after a mean of 4 (± 3.7) months. Two patients were on disability pension before and remained on it after surgery because of ongoing CLBP. Three other patients retired on disability 12 to 24 months after surgery due to CLBP and another for reasons unrelated to spinal problems (systematic lupus).

Some 71% of patients reported satisfaction with the surgery outcome and 89.4% expressed willingness to undergo a new surgery (´definitely´ and ´rather yes´). Both outcome variables were not influenced by a change in ODI. However, the reported lower satisfaction with surgery outcome (´rather no´ and ´definitely not´) and lower willingness to undergo surgery again (´definitely not´) were associated with a lower decrease in VAS after surgery (*p* < 0.05).

## Discussion

Clinical evidence of efficacy for surgery of back pain is lacking. Few randomised controlled trials (RCTs) have been performed because of difficulties in conducting neurosurgical RCTs [26]. Thus, most current NICE guidelines are based on a few prospective RCTs for lumbar fusion or disc arthroplasty. All these RCT studies suffer from methodological flaws. Some RCTs included few patients, some randomised patients to up to three surgical techniques and others offered the same conservative treatment that had previously failed to relieve pain. In addition, there were problems of crossover and loss to follow-up [19, 23]. One of the biggest trials was the Swedish Lumbar Spine Study from 2001. In this study, 294 patients were randomised if the treating surgeon interpreted the pain as emanating from L4/L5 or L5/S1 using the patient’s history, physical examination and radiographic signs. At 2 years, 63% of patients in the surgical group rated themselves as “better” or “much better” compared to 29% of patients in the non-surgical group. However, the authors did not specify which radiographic signs indicated surgical intervention [10]. Although the authors of this study concluded that lumbar fusion could reduce pain and decrease disability in carefully selected patients, this conclusion was not confirmed in Fairbank´s multi-centre RCT [9]. However, in Fairbank’s trial, including 349 participants, the authors reported no information on the diagnostic evaluation of the suspected pain generator and the extent of the surgery. The surgical technique was left to the discretion of the operating surgeon (fusion with or without interbody graft, flexible stabilisation) [9, 23]. Therefore, because of heterogeneity in surgical techniques and vague and differing definitions of the preoperative diagnostic process, NICE has concluded that the evidence for spinal fusion or disc replacement for DDD is poor [19]. Despite the recent survey study among spine surgeons revealing that the majority still perform fusion in patients with CLBP, the use of preoperative diagnostics and tests continues to vary widely [2].

The main problem is simple: the current diagnostic methods and tests cannot unequivocally identify the source of pain in these patients. The history, physical examination and imaging studies cannot identify symptomatic DDD consistently [28]. Discography (instillation of a contrast agent with pain enhancement) or discoblock (instillation of anaesthesia for pain management) can be used to diagnose DDD [7]. Weishaupt et al. found that vertebral endplate abnormalities (Modic changes, MCs) seen on MRI were associated with pain reproduction during discography in patients with CLBP [27]. However, these invasive procedures cannot with certainty select patients with CLBP who would benefit from fusion procedures [28]. Therefore, they are not routinely used in daily practice because of invasiveness, risk of spondylodiscitis and insufficient evidence of their success [4]. Moreover, based on meta-analyses, the relationship between MCs and CLBP is unclear [12] and their presence does not significantly impact the clinical outcomes following lumbar fusion, discectomy or disc replacement [15].

Several studies describing the effectiveness of facet blocks confirmed the high sensitivity and specificity of SPECT/CT imaging for facet syndrome [25]. Brusko et al. published a series of 23 patients operated on for SPECT + spinal degeneration. Of the 23 patients, 9 underwent fusion surgery for cervical discopathy and 14 for lumbar degeneration, with an average of 2.3 levels treated per patient. Eleven patients (47.8%) reported complete remission of symptoms 6 months after the procedure. After 1 year, 82.6% had at least partial improvement of symptoms [3]. Tender et al. also found significant improvement in their 23 patients undergoing lumbar fusion (only 13 for single-level SPECT + DDD). The VAS score for axial pain decreased from 9.35 (± 0.7) preoperatively to 4.87 (± 2.3) 6 months postoperatively [21].

Our study included patients with unclear MRI findings showing multi-level degenerative changes of the lumbar spine. However, it evaluated only cases undergoing single-level fusion for lumbar SPECT + DDD. The objective of this limited assessment was to minimise the bias resulting from the combination of outcomes of single- and multi-level fusions of mixed pathologies (DDDs and facet arthropathies). Moreover, we used only one spinal fusion (TLIF) technique. Although anterior and posterior fusion procedures have similar fusion and complication rates, each has risks and benefits [22]. However, TLIF seems to have the safest profile regarding neural, spinal and vascular events [5]. We believe these facts allow us to achieve the greatest possible generalisability of our results.

Mean VAS decreased from 8.4 (± 1.1) to 3.2 (± 2.5) and ODI from 51.5 (± 7.3) to 20.7 (± 14.68) 2 years after surgery. A 30% reduction in disability and pain (MCID) determines clinically relevant improvement in a broad spine surgery population [1]. Such a reduction was achieved in 84.2% of our patients. Six of our 38 patients (15.8%) did not meet the MCID. One female patient had a significant improvement in ODI but minimal change in VAS score. In the remaining five patients (13.2%), postoperative disability and back pain were similar or even worse than before surgery. The patients showing a lack of satisfactory improvement underwent a CT scan of the lumbar spine during the last visit. All patients exhibited bone growth at the instrumented levels without evidence of pseudarthrosis. Also, an MRI of the lumbar spine performed in these patients did not show significant new pathology requiring surgical treatment. Fritzell et al. reported reduced back pain by 33% and improved ODI by 25% (47 to 36) [10]. Similarly, in Fairbank et al.´s study, the ODI changed from 46.5 (± 14.6) to 34.0 (± 21.1) [9]. As in these studies [9, 10], we also included patients after a previous microdiscectomy or sequestrectomy (5/38 patients) was performed because of radicular pain before the development of severe CLBP. We believe that even such patients may benefit from SPECT/CT examination in case of unclear MRI findings. The greater improvement in our series may be because of the selected cohort of patients with a solitary SPECT + DDD without a more extensive degeneration of the lumbar spine with potential multiple pain generators.

Our early complication rate of 7.9% was lower than in the two large trials (10.8% [9] and 17%[10]). Only two of our patients (5.3%) underwent revision surgery (wound infection and CSF leak). SPECT/CT was negative in 36% of our patients, similar to Tender et al.´s [21] finding of 40% negative examinations. Therefore, in more than one third of patients undergoing diagnostic work-up for CLBP, SPECT/CT may help to guide the patient to manage chronic pain.

Many social, psychological and occupational aspects, as well as the duration of CLBP, are associated with a lower likelihood of returning to work after spine surgery [11]. However, almost 83% of our patients of productive age (except one patient on maternity leave) returned to work after surgery. Therefore, work-related factors should not limit the indication for surgery in these cases. In a Canadian national study of patients undergoing elective spine surgery for degenerative conditions (of which DDD formed only 9.1%), Rampersaud et al. found that satisfaction with surgery outcome was achieved in 84.9% of patients. In the same study, the authors also reported that 5.9% were dissatisfied and 9.2% were neither satisfied nor dissatisfied [20]. Although the satisfaction of our patients was slightly lower (71%) compared to the Rampersaud et al. study, most (89.4%) patients expressed a willingness to undergo a new surgery despite the potential surgical risks.

This study has some strengths. The study is the largest study analysing pain improvement in patients with DDD of the lumbar spine diagnosed by SPECT/CT. We focused exclusively on the one-level SPECT + DDD of the lumbar spine and excluded facet arthropathy and multi-level SPECT + pathologies and findings in more proximal segments of the spine. We used only one technique of spinal fusion (TLIF) and all patients were followed up for 2 years.

However, our study is not without limitations. We did not discuss the cost and radiation exposure of the SPECT/CT imaging procedure. Furthermore, over one third of the examinations were negative. However, SPECT/CT can be considered an additional imaging tool indicated in a select group of patients with CLBP and unclear MRI findings. Moreover, negative patients were diagnosed as non-surgical candidates and were directed to pain management or rheumatology. Another limitation is that there was no control group for comparison. Our results could be confirmed by a multi-centre randomised study evaluating the difference between surgical and maximum conservative treatments on a larger number of patients. We aimed to evaluate only the effect of one-level SPECT + DDD fusion. Therefore, evaluating the conservatively treated SPECT-negative patients or the relationship between multi-level SPECT positivity and the effectiveness of more extensive fusion procedures warrants further study.

## Conclusion

The results of our study demonstrate that one-level fusion for a SPECT/CT positive lumbar DDD results in substantial clinical improvement and self-reported satisfaction with surgical treatment. Consequently, SPECT/CT imaging may be a useful adjunct to differentiate patients with unclear MRI findings who might benefit from surgical treatment.

## Supplementary Information

Below is the link to the electronic supplementary material.

Supplementary file1 (PDF 199 KB)

## Data Availability

All data relevant to the study are included in the article or uploaded as supplementary information.
