# Supplementary material for: Spinal fusion for single-level SPECT/CT positive lumbar degenerative disc disease: the SPINUS I study
Source: Acta Neurochir (Wien). 2023 Jun 22;165(9):2633–40. doi: 10.1007/s00701-023-05666-8 (PMC10477220; doi:10.1007/s00701-023-05666-8)
Supplement: Supplementary file 1 — Supplementary file1 (PDF 199 KB) [file 701_2023_5666_MOESM1_ESM.pdf]

Patient	Age	Sex	BMI	Level	ODI - preOP	ODI 6-months FU	ODI 24-months FU	VAS - preOP	VAS 6-months FU	VAS 24-months FU	MCID	Sick leave pre-OP (months)	Sick leave post-OP (months)	Satisfaction with the effect of OP	Willingness to undergo surgery again	Complications
**1**	40	F	26.8	L5/S1	44	10	4	7	3	0	Yes	6	6	1	1	
**2**	59	M	27.4	L5/S1 *	44	25	20	8	2	2	Yes	0	1	1	1	
**3**	38	M	22.9	L4/5	56	20	16	9	2	0	Yes	3	4	1	1	
**4**	52	M	38.6	L5/S1 *	52	6	6	8	4	4	Yes	12	12	2	2	
**5**	41	F	22.6	L5/S1	48	24	18	10	7	5	Yes	7	2	1	1	
**6**	49	F	22.5	L5/S1 *	42	26	14	10	4	4	Yes	4	5, disability pension 15 months postOP (systemic lupus)	1	1	
**7**	43	F	22.4	L5/S1	48	8	6	8	3	4	Yes	0	8	2	1	
**8**	51	M	23.5	L4/5	40	12	2	7	2	1	Yes	0	1	1	1	
**9**	49	F	19.7	L5/S1	44	32	34	6	6	6	No	0	1	5	5	
**10**	48	F	22.2	L5/S1	42	6	0	8	1	1	Yes	0	6	1	1	
**11**	53	F	29.4	L5/S1	58	14	6	10	0	0	Yes	12	1	1	1	
**12**	33	F	26.7	L5/S1	62	12	16	10	2	5	Yes	6	6	2	1	
**13**	34	F	26.1	L5/S1	52	28	18	8	1	2	Yes	Maternity leave	Maternity leave	1	1	
**14**	63	M	29.4	L3/4	64	32	28	9	7	5	Yes	6	2	1	1	
**15**	45	F	27.5	L5/S1	56	10	6	7	2	2	Yes	0	4	3	1	Deep infection
**16**	44	F	31.1	L5/S1	64	8	4	10	0	0	Yes	5	3	1	1	
**17**	52	F	25.6	L5/S1	56	12	16	10	2	1	Yes	12	3	3	1	
**18**	30	F	20.5	L5/S1	44	8	6	7	2	1	Yes	0	2	1	1	
**19**	54	M	20.2	L2/3	66	36	36	8	6	5	Yes	Disability pension	Disability pension	1	1	
**20**	75	M	24.7	L5/S1	44	6	26	8	1	3	Yes	Old-age pension	Old-age pension	2	1	
**21**	52	F	24.0	L5/S1	48	4	4	7	0	1	Yes	0	8	1	1	
**22**	56	F	24.2	L3/4	58	24	18	8	6	6	No	0	16	4	4	
**23**	45	F	26.1	L5/S1	46	66	50	8	7	5	No	9	24, followed	2	1	

													by disability pension			
**24**	44	F	20.2	L5/S1 *	64	20	28	10	3	4	Yes	7	1	3	2	
**25**	49	F	24.8	L5/S1	52	32	30	7	4	5	Yes	0	1	1	1	
**26**	27	F	30.5	L4/5	44	2	6	9	1	0	Yes	12	2	1	1	
**27**	43	M	37.0	L5/S1	52	4	8	7	0	0	Yes	0	2	1	1	
**28**	71	M	35.5	L2/3	48	26	20	9	6	5	Yes	Old-age pension	Old-age pension	3	4	
**29**	46	F	32.1	L5/S1 *	56	34	28	9	5	5	Yes	Disability pension	Disability pension	2	1	
**30**	63	M	35.6	L2/3	48	6	18	8	0	0	Yes	0	3	2	1	
**31**	47	F	29.3	L5/S1	46	10	26	7	1	3	Yes	4	12	2	1	
**32**	63	M	29.0	L4/5	52	18	30	9	3	4	Yes	4	1	1	1	
**33**	42	F	29.0	L5/S1	46	50	54	9	8	8	No	3	20, followed by disability pension	4	2	Lumbar swelling
**34**	48	F	26.0	L5/S1	48	36	16	8	4	0	Yes	0	3	1	1	
**35**	32	F	26.0	L5/S1	56	24	38	10	4	5	Yes	18	3	4	1	
**36**	46	F	22.5	L4/5	44	40	36	8	8	8	No	0	6	3	5	CSF leak, wound infection
**37**	46	M	26.5	L4/5	64	12	42	10	2	6	Yes	5	3	3	2	ASD L3/4 and DH L2/3 16M after surgery
**38**	52	M	26.2	L1/2	58	50	52	9	7	7	No	12	12, followed by disability pension	4	1	

Appendix: Patient-level data. M – male, F – female, * – previous discectomy, VAS – visual analogue scale, ODI – Oswestry Disability Index, MCII – minimum clinically important difference (30% reduction in VAS and ODI). ASD – adjacent segment disease, DH – disc herniation. Satisfaction with the effect of surgery and willingness to undergo surgery again: 1 – definitely, 2 - rather yes, 3 – maybe, 4 - rather no, 5 - definitely not.
